# The benefits of Tai Chi and brisk walking for cognitive function and fitness in older adults

**DOI:** 10.7717/peerj.3943

**Published:** 2017-10-20

**Authors:** Zhiguang Ji, Anmin Li, Tian Feng, Xiaolei Liu, Yihong You, Fanying Meng, Ruoqing Wang, Jialing Lu, Chunhua Zhang

**Affiliations:** 1 School of Kinesiology, Shanghai University of Sport, Shanghai, China; 2 Department of Traditional Sports, Beijing Sport of University, Beijing, China

**Keywords:** Tai Chi, Brisk walking, Executive function, Older adults, Fitness

## Abstract

The purpose of this study was to investigate the benefits of exercises with different cognitive demands for cognitive functions (Executive and non-Executive) in healthy older adults. A cross-sectional design was adopted. In total, 84 healthy older adults were enrolled in the study. They were categorized into the Tai Chi group (TG), the brisk walking group (BG) or the control group (CG). Each participant performed the Stroop task and a digit comparison task. The Stroop task included the following three conditions: a naming condition, an inhibition condition and an executive condition. There were two experimental conditions in the digit comparison task: the non-delay condition and the delay condition. The results indicated that participants of the TG and BG revealed significant better performance than the CG in the executive condition of cognitive tasks and fitness. There was no significant difference of reaction time (RT) and accuracy rate in the inhibition and delay conditions of cognitive tasks and fitness between the TG and BG. The TG showed shorter reaction time in the naming and the executive conditions, and more accurate in the inhibition conditions than the BG. These findings demonstrated that regular participation in brisk walking and Tai Chi have significant beneficial effects on executive function and fitness. However, due to the high cognitive demands of the exercise, Tai Chi benefit cognitive functions (Executive and non-Executive) in older adults more than brisk walking does. Further studies should research the underlying mechanisms at the behavioural and neuroelectric levels, providing more evidence to explain the effect of high-cognitive demands exercise on different processing levels of cognition.

## Introduction

The consequences of old age, including cognitive decline, has been an essential problem around the world (Kurth, Cherbuin & Luders, 2017). The decline in cognitive function, especially executive function, is impacted by ageing (Colcombe & Kramer, 2003). Executive function refers to an established higher-order cognitive process of control and coordination that allows for behavioural adaptation to complex or novel situations (Diamond, 2012). In particular, executive function consists of three sub-constructs: inhibition control, working memory, and task-switching (Diamond, 2012; Miyake et al., 2000).

Research has indicated that a positive relationship exists between physical activity and executive function (Salthouse, 2010). Though most of previous exercise-cognition researches have focused on aerobic exercises (e.g., brisk walk, jogging and treadmill), later studies have demonstrated resistance exercise (Chang et al., 2012), dance (Chuang et al., 2015; Kosmat & Vranic, 2016) or coordination training (Voelcker-Rehage & Niemann, 2013) also could delay the decline of cognitive function. Many researchers have investigated the benefits of Tai Chi or aerobic exercises on cognitive decline, but limited studies have compared the benefits of aerobic exercises and Tai Chi on executive function. To our knowledge, few studies compared aerobic exercise and Tai Chi (Fong et al., 2014; Taylor-Piliae et al., 2010). Taylor-Piliae et al. (2010) revealed the results showed Tai Chi resulted in greater improvements in cognitive function compared to the attention-control groups and Western exercises, which included aerobic exercise, incorporated endurance, resistance/strength, and flexibility exercises. Conversely, another study by Fong et al. (2014) compared the task-switching aspect of executive function between participants undergoing aerobic exercises and Tai Chi participants. The results showed a positive relationship was observed regardless of the types of physical activity and task-switching (Fong et al., 2014). Some researchers suggested TC practice might have a similar pattern to the cortical morphology associated with aerobic exercise (Wei et al., 2013). In ERP studies, Tai Chi and aerobic exercise groups had a significantly larger P300 amplitude compared with sedentary group in cognitive tasks, but no difference was found between Tai Chi and endurance exercise groups (Dai et al., 2013; Fong et al., 2014). However, most of those studies did not assess the cardiorespiratory fitness or other physical fitness, so they did not control several confounding variables, which may affect cognition. Furthermore, the inhibition, working memory, and task-switching which are predominant sub-constructs of executive function need be reviewed. Only one of the sub-constructs was mentioned in previous studies. Therefore, this study should compare the effect on cardiorespiratory fitness and cognitive functions between Tai Chi and aerobic exercise.

Tai Chi, which is a moderate-intensity high demand activity that combines memorized, controlled physical postures and movements (Miller & Taylor-Piliae, 2014), is a high cognitive demand exercise. Chang et al. (2010) thought that the intensity of Tai Chi was similar to brisk walking and safe for older adults. Therefore, the difference between Tai Chi and brisk walking was the intensity of cognitive demand. Notably, studies found open-skill (i.e., table tennis and tennis) and closed-skill (i.e., jogging and swimming) exercises induced differential effects on executive functions. Results of studies supported that open-skill exercise may have a more beneficial effect on executive function in older adults (Dai et al., 2013; Guo et al., 2016; Huang et al., 2014). These studies found compared to closed-skill exercises, open-skill exercises is high cognitive demanded exercise that require a quick response to deal with complex environments, and more cognitive resources must be demanded to increase the efficiency of decision-making processes (Dai et al., 2013; Guo et al., 2016). In line with it, Pesce (2012) argued that future research should emphasize the qualitative aspects of exercise, such as exercise involving more physical and exercise-related cognitive features, rather than only targeting quantitative exercise characteristics such as exercise intensity, duration, and frequency. Therefore, cognition demand of exercise may one of potential influential factors of the effect of exercise on cognitive function. However, these studies did not compare several confounding variables in different exercises, such as cardiorespiratory fitness, they could not prove the benefit of exercises with different cognitive demands on cognitive functions.

The type of cognition has been examined from basic information processing to higher levels of cognition (i.e., executive function). Some studies revealed that exercise has a different impact on different processing levels of tasks. Previous studies indicated that long-term exercise revealed the strongest effect on the executive function of older adults and formulated the selective improvement hypothesis (Colcombe & Kramer, 2003; Kramer et al., 1999). Later studies supported this theory, demonstrating aerobic exercise is more likely to benefit performance on tasks that involve higher rather than lower processing levels, especially in the executive function (Dupuy et al., 2014; Mekari et al., 2015). So, what is the effect of Tai Chi on different processing levels of task? In the reviews, Tai Chi improved executive function in older adults (Zheng et al., 2015). However, the results of different processing levels of tasks were inconsistent. For example, some studies revealed that Tai Chi could improve the performance in TMT-A and TMT-B tests (Hung & Andreas, 2012; Reid-Arndt, Matsuda & Cox, 2012). In contrast, Matthews & Williams (2008) found TC group improved in TMT-B, but no improvement in TMT-A. TMT-A and TMT-B tests could be classified into different processing levels of tasks, such as attention and executive function by domain (Smith et al., 2010). Thus, the conclusion that Tai Chi was more beneficial for tasks that relied on executive control processes was still open to debate. Other results revealed that Tai Chi can also improve cognition like attention and memory functions, and not only executive control (Kim et al., 2016; Man, Tsang & Hui-Chan, 2010). Taken together, the current study was to compare the performance on different tasks which can identify the different cognitive processing between Tai Chi and aerobic exercise. If different processing levels of tasks could be explicitly assessed, it would help researchers to normalize the measurement and facilitate the comparisons for future research. In these studies, only the TMT task was used to measure the different processing levels of cognitive function. Based on previous research, two types of tasks have been applied to assess the performance in different cognitive demand: an executive task and a non-executive task (Dupuy et al., 2014; Gauthier et al., 2015).

Accordingly, the main purpose of this study was to assess the benefits of exercises with different cognitive demands (Tai Chi and brisk walking) on cognitive functions (Executive and non-Executive) and fitness in healthy older adults. The classifications of these tasks according to their processing levels and their main brain regions were relevant. Therefore, the tasks involved higher mental functioning capacity by the frontal lobe and lower by the frontal lobe (Voelcker-Rehage & Niemann, 2013). We hypothesized that: (1) Tai Chi and brisk walking exercisers would show shorter RTs and more accurate in the executive function condition of the Stroop task and the digit comparison task than sedentary controls, (2) Tai Chi exercisers would show shorter RTs and more accurate in the non-executive function condition of the Stroop task and the digit comparison task than brisk walking exercisers and sedentary controls, and (3) Tai Chi exercisers would exhibit similar cardiorespiratory fitness and dynamic balance ability as brisk walking exercisers, but better than sedentary controls.

## Methods

### Ethical approval

The study was conducted ethically and received approval from the Ethics Committee of Shanghai University of Sport (No. 2017032).

### Participants

Healthy older adults aged 60 to 72 years from a local community in Shanghai, China were recruited via advertisements placed throughout the community. The inclusion criteria were as follows: no history of major psychiatric illness (e.g., schizophrenia or bipolar disorder) or other serious neurological or musculoskeletal diagnoses; score ≥26 on the Mini-Mental Status Examination (MMSE); right-handedness; and normal or corrected-to-normal vision. Additionally, these participants met the following fitness criteria: no history of falling; the ability to walk without a cane or other assistive devices; body mass index (BMI) <25.0 and >18.5. All participants signed the written informed consent form provided by the Ethics Committee of Shanghai University of Sport.

A total of 84 older adults who met the requirements were recruited for the formal experiments. The eligible participants were assigned to three groups: the Tai Chi group (TG), the brisk walking group (BG), and the control group (CG). Each group included 28 participants. Potential subjects who engaged in both types of physical exercises or had practised for fewer than five years were excluded. Participants who had little or no exercise training for at least five years were assigned to the control group. The Tai Chi and brisk walking groups generally participated in Tai Chi (i.e., Tai Chi chuan or Qi Gong) or brisk walking exercises at least five times per week for 30 min per session for at least five years. The type-of-exercise questionnaire evaluated the specific type of exercise performed and the frequency per week, the duration per session and the number of years the participant had been practicing the specific type of exercise.

### Experimental procedure

The participants visited the laboratory individually two times. At the first visit, the participants were given a general description of the study, signed the consent form, and completed various questionnaires (a health history and demographics questionnaire; the International Physical Activity Questionnaire (IPAQ), a type-of-exercise questionnaire; and the MMSE) under the instruction of the experimenter. After the participants’ eligibility for participation in the study was confirmed, they completed the fitness tests. Within three days, the participants visited the laboratory again to complete the cognitive tests. Prior to each task, the participants performed 20 practice trials with feedback for each condition. They continued with the experiments only if their accuracy rate reached 75% in the practice trials. The order of the two tasks was counterbalanced within the groups.

### Anthropometrics and fitness

Height (m) and body weight (kg) were evaluated to determine the BMI (kg/m^2^). Hand-grip strength was measured with a hand-held dynamometer (Sammons Preston Rolyan; Jamar, Nottinghamshire, UK). The participants performed the hand-grip test twice with each hand, and the best results were used for the analysis. Physical activity levels were assessed with the Taiwan version of the IPAQ (Liou et al., 2008). The short version of the IPAQ is an 8-item scale that estimates the number of minutes spent engaging in vigorous and moderate intensity activity, walking, and sedentary behaviour during the previous seven days. The total amount of physical activity is calculated by adding the MET minutes and categorizing them as inactivity (i.e., fewer than 600 METs), moderate activity (i.e., 600–3,000 METs), and sufficient activity (i.e., >3,000 METs overall).

The 6-minute walk test (6MWT) is a simple test that is widely used in clinical settings to assess cardiorespiratory fitness (Burr et al., 2011; Camarri et al., 2006). Prior to the 6MWT evaluation, all participants completed a practice session. The 6MWT was performed over a 50-m-long straight course within an enclosed, level corridor. In both settings, each end of the 10-m walking course was marked on the floor using coloured tape. The participants were instructed to “cover as much distance as possible” during the test. The number of metres walked was recorded.

The timed up-and-go (TUG) test was used to evaluate dynamic balance ability (Clark et al., 2010; Porciuncula, Rao & Mcisaac, 2016). The participants were given verbal instructions to stand up from a seated position in a chair, walk a distance of 3 m as quickly and safely as possible, cross a mark on the floor, turn around, walk back to the chair and sit. The participants were allowed one TUG practice trial to familiarize themselves with the tasks. The study selected the following two conditions for the dual-task TUG: (1) in the mTUG task, the participants were asked to remember six random numbers while walking and to verbally repeat the correct numbers after sitting down, and (2) in the cTUG task, the participants were asked to cross a 17-cm-high (kerb height) obstacle while walking. The tests were completed three times, and the shortest duration was recorded for each task (Shumway-Cook, Brauer & Woollacott, 2000). The participants were instructed to walk as quickly as possible and to perform the secondary task as quickly and/or accurately as possible. For safety reasons, the participants practised all the tasks. Furthermore, the participants did not complete the test alone; an experimenter supervised the test throughout the experiment.

### Cognitive test

#### Modified stroop task

**Figure 1:**
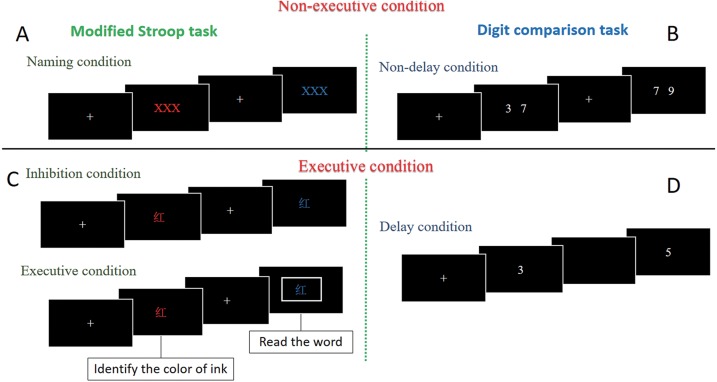
Illustration of the modified stroop task and digit comparison task. (A) The naming condition of the Modified Stroop task; (B) The non-delay condition of the digit comparison task; (C) The inhibition and executive conditions of the Modified Stroop task; (D) The non-delay condition of the digit comparison task.

The computerized modified Stroop task is based on the Stroop task and includes the following three conditions: a naming condition (non-executive), a inhibition condition, and an executive condition (Bohnen, Twijnstra & Jolles, 1992; Olivier et al., 2015). Each condition includes two blocks, and each block is composed of 30 trials, resulting in 180 trials for the three experimental conditions. All the experimental trials began with a fixation cross for 1,000 ms, and all visual stimuli appeared at the centre of the computer screen for a duration of 2,000 ms. The participants provided their responses by pressing the “F” button on a QWERTY keyboard with their left index finger if the testing stimulus was “Blue” and pressing the “J” button with their right index finger if it was “Red”. In the naming condition, a visual stimulus (XXX) coloured red or in blue was presented, and the participants were asked to identify the colour of the writing by pressing a button. One block of the naming condition had 30 trials consisting of 15 red stimuli and 15 blue stimuli with mixed presentation. In the inhibition condition, the participants were presented with the word 蓝 in red or 红 in blue, and they had to restrain from reading the word and respond only to the text colour. One block of the inhibition condition had 30 trials consisting of 15 congruent and 15 incongruent trials. Two trial conditions were included in the executive condition task: inhibition and switch. Half of the trials were the same as those described for the inhibition condition. In the other trials, the words presented were surrounded by a rectangle (i.e., the word “蓝” was printed in red ink), and when a rectangle appeared around the word, the participants were instructed to change their response to read the word, ignore the colour of the writing, and respond to the word itself (i.e., 蓝). Thirty trials (20 inhibition and 10 switch) were included in one block of the executive condition (see Figs. 1A, 1C). The experimental blocks were interspersed with a 1-min condition called “Rest”. The total Stroop task duration was approximately 15 min.

#### Modified digit comparison task

Working memory was evaluated using the modified digit comparison task. The participants were informed to indicate which number was larger by pressing the button. The number was represented by a pair of digits was ranging from 0 to 9 (e.g., “7” and “9”). There were two experimental conditions: non-delay (non-executive) and delay (executive) (Turner & Spreng, 2012). Each run of the task involved two blocks of each condition composed of 30 trials in each block, resulting in 60 trials per experimental condition. In the non-delay condition trials, the visual stimulus set consisted of a pair of numbers that were simultaneously presented on the computer screen. The participants were required to press the “F” button on a QWERTY keyboard with their left index finger if the left number was larger and the press button “J” with their right index finger if the right number is larger. In the delay condition trials, there was a 1,500-ms delay between the digit pairs. Participants provided their responses by pressing the “F” button on a QWERTY keyboard with their left index finger if the first number was larger, and the “J” with their right index finger if the later number was larger. Experimental blocks where interspersed with a 1-min condition named Rest. Total digit comparison task duration was approximately 10 min (see Figs. 1B, 1D).

### Statistical analyses

The statistical analyses were performed using SPSS version 22. One-way ANOVA was used to determine statistically significant differences in age, weight, height, BMI, the MMSE score, education, grip strength, 6MWT and the IPAQ score among the three groups. A mixed design 3 (Group: Tai Chi, brisk walking, and sedentary) × 3 (Condition: TUG, cTUG, and mTUG) ANOVA was used to analyse duration differences for the TUG test. RT and accuracy rate were used to evaluate the performance of cognitive tests. The RT data (i.e., the elapsed time from when the test stimuli were presented to when the keys were pressed) and accuracy rate data were collected using E-Prime (version 1.1) software. All error trials and trials with RTs more than three standard deviations from the individual mean were treated as outliers and were discarded prior to the analysis. Two mixed-design 3 (Group: Tai Chi, brisk walking, sedentary) × 3 (Condition: naming, inhibition, executive) ANOVAs were used to analyse differences in the RT and accuracy rates for the Stroop task. Two mixed-design 3 (Group: Tai Chi, brisk walking, and sedentary) × 2 (Condition: delay and non-delay) ANOVAs were used to analyse differences in the RT and accuracy rates for the digit comparison task. Post hoc comparisons were indicated using least significant difference tests. Effect size was expressed as partial eta-squared (*η*^2^) to determine the magnitude of the effect when significant main and interaction effects were reached. The significance threshold was set at *p* < 0.05.

## Results

### Anthropometrics and fitness

No significant differences were found in age (*F*_(2,81)_ = 0.70, *p* = 0.50), weight (*F*_(2,81)_ = 0.54, *p* = 0.59), height (*F*_(2,81)_ = 0.62, *p* = 0.54), BMI (*F*_(2,81)_ = 0.57, *p* = 0.57), MMSE score (*F*_(2,81)_ = 0.08, *p* = 0.93), or education (*F*_(2,81)_ = 0.70, *p* = 0.51) between the groups. There were significant differences in grip strength (*F*_(2,81)_ = 3.57, *p* < 0.05, *η*^2^ = 0.08), 6MWT (*F*_(2,81)_ = 165.72, *p* < 0.001, *η*^2^ = 0.80), and IPAQ scores (*F*_(2,81)_ = 177.85, *p* < 0.001, *η*^2^ = 0.82). The post hoc comparison showed that the TG and BG had greater grip strength, longer 6 MWT and higher IPAQ scores than the CG, but no significant difference was found between the TG and BG. There were significant main effects of the task (*F*_(2,162)_ = 114.18, *p* < 0.001, *η*^2^ = 0.59) and group (*F*_(2,81)_ = 8.26, *p* < 0.001, *η*^2^ = 0.17). No significant interaction was found between the task and the group (*F*_(4,162)_ = 1.824, *p* = 0.13). The post hoc comparison revealed that TG and BG were shorter than CG regardless of conditions in TUG test. Table 1 shows the main characteristics of the subjects in the different groups.

**Table 1:** Participant characteristics.

	Tai Chi group (28)	Aerobic group (28)	Control group (28)
Age, years	66.14 ± 3.40	65.36 ± 4.06	65.04 ± 3.33
Gender (women, *n*)	18	19	19
Weight, kg	59.68 ± 5.29	57.75 ± 7.09	58.75 ± 8.24
Height, cm	163.29 ± 5.68	162.00 ± 6.53	161.43 ± 6.89
BMI	22.39 ± 1.76	21.95 ± 1.78	22.51 ± 2.62
MMSE score	27.93 ± 0.66	27.93 ± 0.94	27.86 ± 0.80
Education, years	11.11 ± 1.85	11.14 ± 1.76	10.64 ± 1.68
Muscle strength, kg	28.83 ± 6.51	28.47 ± 5.62	25.08 ± 5.16^*^^,^^#^
6MWT, m	573.41 ± 13.08	575.94 ± 14.12	516.09 ± 14.50^*^^,^^#^
IPAQ, METs	2453.39 ± 486.03	2415.88 ± 610.81	426.84 ± 159.71^*^^,^^#^
TUG, s	6.03 ± 0.45	6.26 ± 0.68	6.68 ± 0.58^**^^,^^##^
cTUG, s	6.95 ± 0.54	6.96 ± 0.64	7.30 ± 0.67^*^
mTUG, s	6.29 ± 0.50	6.48 ± 0.55	7.03 ± 0.68^**^^,^^##^

**Notes.**

BMI body mass index MMSE min mental state exam IPAQ International physical activity questionnaire METs metabolic equivalents 6MWT six minute walk test TUG Time UP and GO cTUG TUG with crossing obstacle mTUG TUG with memory task

* Represent the significant differences between the Tai Chi group and the control group, *p* < 0.05.

** Represent the significant differences between the Tai Chi group and the control group, (*p* < 0.01).

# Represent the significant differences between the brisk walking group and the control group, *p* < 0.05.

## Represent the significant differences between the brisk walking group and the control group, *p* < 0.01.

### Modified stroop task

Regarding the RT results, there were main effects for condition (*F*_(2,162)_ = 624.07, *p* < 0.001, *η*^2^ = 0.89), and group (*F*_(2,81)_ = 30.58, *p* < 0.01, *η*^2^ = 0.44). An interaction between condition and group was observed (*F*_(4,162)_ = 10.58, *p* < 0.001, *η*^2^ = 0.21). The post hoc comparison of RT in two conditions showed that RT in the non-delay condition was shorter than that in the delay condition for three groups (*p* < 0.05). Moreover, regarding the groups, the TG had a shorter RT than the CG (*p* < 0.001) and the BG (*p* < 0.05) in the naming condition, but no significant difference was found between the BG and CG (*p* = 0.11). In the inhibition condition, the TG (*p* <0.001) and BG (*p* < 0.001) had shorter RTs than the CG, but no significant difference was found between the TG and BG (*p* = 0.10). In the executive condition, the TG had a shorter RT than the BG (*p* < 0.001) and the CG (*p* < 0.001), and the BG had a shorter RT than the CG (*p* < 0.001).

**Figure 2:**
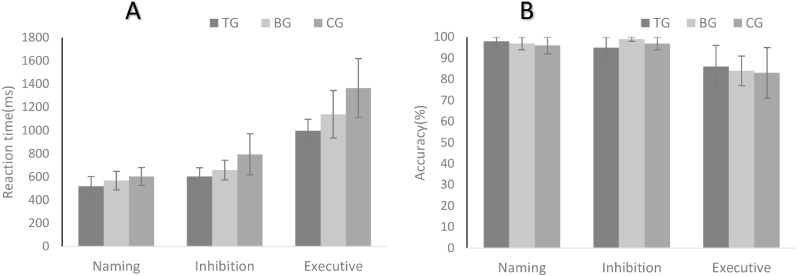
Reaction time (A) and accuracy rates (B) in the modified Stroop task: the naming condition, the inhibition condition and the executive condition. TG, Tai Chi group; BG, brisk walking group ; CG, control group.

For accuracy rate, there was a significant main effect of group (*F*_(2,162)_ = 45.74, *p* < 0.001, *η*^2^ = 0.36), but no significant main effect of condition (*F*_(2,81)_ = 0.92, *p* = 0.40). No interaction between condition and group was observed (*F*_(4,162)_ = 1.11, *p* = 0.35). The post hoc comparison showed that accuracy rate in the non-delay condition was greater accuracy than that in the delay condition for three group (*p* < 0.01). For the effect of group, the TG exhibited greater accuracy than the CG (*p* < 0.05) in the naming condition, but no significant differences between the TG and the BG (*p* = 0.56) or between the BG and the CG (*p* = 0.12) were found. In the inhibition condition, the TG had greater accuracy than the BG (*p* < 0.05), but no significant differences were found between the TG and the CG (*p* = 0.14) or between the BG and the CG (*p* = 0.27). In the executive condition, there were no significant differences among the three groups. All results are presented in Fig. 2.

### Modified digit comparison task

The results of the modified digit comparison task in the non-delay and delay conditions are presented in Fig. 2. There were significant main effects of condition (*F*_(1,81)_ = 189.98, *p* < 0.001, *η*^2^ = 0.70) and group (*F*_(2,81)_ = 189.98, *p* < 0.001, *η*^2^ = 0.03) and a significant interaction between condition and group (*F*_(2,81)_ = 28.46, *p* < 0.001, *η*^2^ = 0.41). The post hoc comparisons of condition revealed that RT in the naming condition and inhibition condition was shorter than that in the executive condition for three groups (*p* < 0.01). There was no significant difference of the RT between the naming condition and inhibition condition in the BG and CG. In the TG, RT in the naming condition was shorter than that in the inhibition condition (*p* < 0.05). The results of group effects showed that the TG had a shorter RT than the BG (*p* < 0.001) and the CG (*p* < 0.001), and the BG had a shorter RT than the CG (*p* < 0.05). In the non-delay condition, no significant differences were found among the three groups.

**Figure 3:**
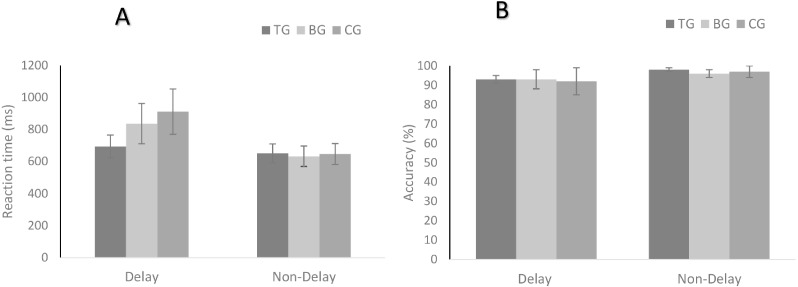
Reaction time (A) and accuracy (B) in the modified digit comparison task: the non-delay condition and the delay condition. TG, Tai Chi group; BG, brisk walking group; CG, control group.

There was a main effect of the accuracy rate of the conditions (*F*_(1,81)_ = 39.86, *p* < 0.001, *η*^2^ = 0.33). There was no main effect of groups (*F*_(2,81)_ = 0.67, *p* < 0.52), and no significant interaction was observed between the conditions and the groups (*F*_(2,81)_ = 0.57, *p* = 0.57). Post hoc comparisons revealed that the higher accuracy rate was showed in non-delay condition (*p* < 0.05). All results are presented in Fig. 3.

## Discussion

This study aimed to compare the differences between the effects of Tai Chi and brisk walking on the cognitive functions of inhibition and working memory as well as the cardiorespiratory fitness and dynamic balance ability in healthy older adults. Consistent with previous studies, the older adults who engaged in exercise, regardless of the exercise type, showed shorter RT than the age-matched sedentary controls in executive condition. Due to the high cognitive demands of the exercise, the TG benefit cognition on both non-executive and executive condition of cognitive tasks more than the CG and the BG does. Additionally, the TG and BG showed better cardiorespiratory fitness and dynamic balance ability than the CG, but no significant difference was found between the TG and BG.

Based on previous research, we used cognitive tasks involving two processing levels: executive and non-executive (Dupuy et al., 2014; Gauthier et al., 2015). A significant main effect of condition was observed in terms of longer RTs for the executive function conditions than the non-executive function conditions of the Stroop and digit comparison tasks, replicating previous studies in which executive condition tasks required greater amounts of executive control. Mekari et al. (2015) concluded that the results for the naming condition of the Stroop task was similar to those for simple reactions that did not require a substantial executive control component. A previous functional magnetic resonance imaging study demonstrated the presence of different cerebrovascular reactivities between executive and non-executive (naming) condition of the Stroop task (Gauthier et al., 2015). The task-related regions in the frontal area of the brain exhibited significantly increased brain activity during the executive condition compared with the naming condition of the Stroop task (Dupuy et al., 2014).

The most important purpose of the present study was to indicate the relationship between the type of exercise and the executive function, especially for the Tai Chi and brisk walking groups. In the present study, the TG and BG exhibited significantly shorter RTs in the inhibition condition, the executive condition of the Stroop task and the delay condition of digit comparison task than the CG. Moreover, the older adults in the TG and BG showed similar or greater accuracy rates compared with the CG, indicating that the shorter RTs could be attributed to the physical activity rather than to a speed-accuracy trade-off. The results are consistent with previous studies that both Tai Chi and aerobic exercise can improve inhibition and working memory (Chang et al., 2013; Walsh et al., 2015) in older adults. Previous meta-analyses have confirmed that Tai Chi has moderate effects on verbal working memory which was measured by Digit span test and the executive function which was measured by TMT (Wu et al., 2013). Furthermore, the BG was significantly correlated with shorter RTs in the executive condition but not in the non-executive condition. The present results supports the hypothesis that selective improvement due to long-term aerobic exercise is more beneficial for tasks that require executive control than for tasks that do not (Colcombe & Kramer, 2003; Kramer et al., 1999). In addition, fitness, a multi-faceted concept that includes physical (i.e., cardiovascular fitness and muscular strength) and motor fitness (i.e., flexibility, speed, balance and fine coordination) (Brent, 2010) have been proposed as potential factors that promote healthy cognitive aging (Miller et al., 2012). Researches suggested that a higher physical activity level and cardiorespiratory fitness are positively associated with executive function in older adults (Fong et al., 2014; Huang et al., 2014; Voelcker-Rehage & Niemann, 2013; Zimmerman et al., 2014). In current study, the TG and BG both had higher physical activity levels than the CG. Consistent with previous results, higher levels of physically activity were correlated with better executive function in older adults (Chang et al., 2013). Similarly, the results suggest that brisk walking and Tai Chi can both improve cardiorespiratory fitness and dynamic balance ability (Van Iersel et al., 2008), but no difference between the TG and BG. These results were consistent with the previous study indicated that higher cardiorespiratory fitness was associated with better performance on executive function tasks (Dupuy et al., 2014). Additionally, we sought to determine whether Tai Chi can improve cognitive function without sacrificing physiological functions. In the current study, the results showed that the TG and BG showed shorter reactive times than the sedentary older adults in the TUG and mTUG tests. This result also proved that TG and BG showed better cognitive function than CG because, especially in the mTUG test, a decreased gait ability during dual tasks could be due to a limited capacity to process attention or to competition for cognitive resources (Mirelman et al., 2012). The TG and BG may conserve their cognitive resources and pay more attention to walking. The results demonstrated that Tai Chi and brisk walking can produce greater dynamic balance ability and cognitive function, which was consistent with our hypothesis and previously published studies (Hakim et al., 2004; Lee et al., 2012; Zhang et al., 2014).

The interesting result was with the different effect on cognitive function between the TG and BG. In current study, older adults in the TG performed better in the naming condition and executive condition than BG. The reasons for these different effects on cognitive function between TG and BG are remain unclear. However, the different characteristics between Tai Chi and brisk walking provide evidence. Tai Chi and brisk walking are both moderate-intensity activities that offer cognitive benefits (Chang et al., 2010), but there are different cognitive demands between them. Zheng et al. (2015) reported that it involves learning and memorizing new skills and movement patterns and sustaining attention, which could be helpful for improving working memory, divided attention and overall executive ability. Taken together, these elements suggest that, compared with brisk walking, the benefits of Tai Chi may be attributed to the increased cognitive demands of practising the exercise. Lauenroth, Ioannidis & Teichmann (2016) found that training that utilized combined cognitive exercise and aerobic training interventions improved attention, processing speed and executive function to a greater extent than aerobic exercise alone. The results were in accordance with studies of Tai Chi. Kim et al. (2016) noted that Tai Chi could improve attentional inhibition assessed by the Antisaccade task and mental attention measured using the Figural Intersections Task. Therefore, current results can support that Tai Chi could improve the performance in tests which examined from basic information processing to higher levels of cognition (Hung & Andreas, 2012; Reid-Arndt, Matsuda & Cox, 2012). A systematic review showed that Tai Chi exercise benefited attention, memory and perception in healthy adults (Zheng et al., 2015). In contrast, evidence from previous studies suggests that aerobic exercises that have little or no cognitive demand can have a beneficial effect on tasks that rely heavily on executive functions (Fabre et al., 2002; Smiley-Oyen et al., 2008). Diamond (2011) stated that older individuals should participate in exercises that have a higher cognitive demand rather than in a single, highly automated aerobic exercise (running on a treadmill, riding a stationary bike, or walking quickly) to improve their cognitive function. However, the results showed no difference of RT between TG and CG on non-executive of the digit comparison task. As an explanation, though the naming condition of the Stroop task and non-delay condition of the digit comparison task are both non-executive condition, they may apply to assess the different aspect of cognition. Additionally, the digit comparison task may rely on more school knowledge than the naming condition of the Stroop task. Due to the fact that no significant difference in years of education was found between the TG, BG and CG, the RT did not vary in all groups. The results implied that the “selective improvement” may exist not only between executive and non-executive function, but also in different aspect of cognitive function.

This study had some limitations that should be considered in future investigations. First, the sample sizes of the participant groups were small. Larger sample sizes may enhance our current findings. Second, the intensity of brisk walking and Tai Chi was measured using questionnaires in current study. Further studies could perform objective measurements using devices such as accelerometers. Third, the relationship between different exercise types and executive functions can be revealed in a cross-sectional design, but this design type could not explain the mechanisms underlying the improvement. Therefore, intervention studies are needed in the future to support the results. Lastly, although Tai Chi exercises have higher cognitive demands than highly automated types of exercise, the cognitive effort required differed across the participants. The amount of attentional resources devoted to the Tai Chi practice may affect the exercises benefits regarding executive function. This study contributes to the literature on the relationship between exercise types and executive function in older adults. The results suggest that future related research should consider that practice years and the automation levels of motor skills may influence the effects of exercise on cognition.

## Conclusions

This study found that older adults who participated in either brisk walking or Tai Chi exhibited better executive function and fitness than sedentary older adults. Furthermore, Tai Chi was found to have a greater beneficial effect on cognitive functions (Executive and non-Executive) than brisk walking, a finding that may be related to the high cognitive demands of Tai Chi. The implication of this finding is that exercises with high cognitive demands provide greater benefits in terms of maintaining and improving cognitive function, although their effects on physical fitness levels were similar to the effects of aerobic exercise. Further studies should research the underlying mechanisms at the behavioural and neuroelectric levels, providing more evidence to explain the effect of high-cognitive demands exercise on different processing levels of cognition.

## Supplemental Information

Supplemental Information 1 Raw data
